# People Who Inject Drugs in Mozambique: We need to normalize HIV treatment and care services in specialized community centers for people who inject drugs!

**DOI:** 10.1186/s12954-023-00910-x

**Published:** 2024-01-06

**Authors:** Cynthia Semá Baltazar, Auria Ribeiro Banze, Jessica Seleme, Makini Boothe

**Affiliations:** 1 National Institute of Health, P.O. Box 264, Maputo, Mozambique; 2 Mozambique National Program for STI, HIV and AIDS Control, Ministry Oh Health, Maputo, Mozambique; 3 UNAIDS, Maputo, Mozambique

**Keywords:** People who inject drugs, Treatment, HIV, Mozambique

## Abstract

Globally, People Who Inject Drugs (PWID) have limited healthcare, treatment, and prevention services, and they frequently experience stigma and negative attitudes toward healthcare providers when accessing services. Mozambique, with a general population HIV prevalence of 12.5%, has one of the highest rates in the world, and the PWID population has the highest HIV prevalence among key populations, estimated at nearly 50%. Less than half of HIV positives who inject drugs are linked to HIV treatment and are retained in care. One of the main reasons is that HIV treatment is mainly provided in a public health facility and PWID delayed accessing healthcare since they anticipated mistreatment from multiple levels of healthcare providers. To improve the health outcomes in this group, we need to treat them where they feel comfortable and respected. In this commentary, we outline the importance of innovative approaches to enhance the management of HIV-positive PWID. As a country gets close to controlling the HIV epidemic, refocusing and targeting responses to the highest-risk groups becomes even more essential for shaping more effective HIV interventions and achieving epidemic control.

## Background

The recent Global Health Strategy for the HIV response (2021–2026) sets a framework for moving toward the goal of ending AIDS as a public health threat by 2030. The strategy emphasizes the adoption of an inequalities lens that ensures that the gains in the HIV Response are equitable and experienced by key and vulnerable populations. This requires that the new comprehensive set of targets must be applied to all groups. Foremost among this framework for measuring progress are indicators related to HIV services (95-95-95), service integration, and targets aimed to remove societal and legal barriers to accessing services [1].

It is undoubted that injection drug use plays a significant role in driving HIV epidemics with negative health consequences in many countries around the world [2–4]. Globally, nearly 15 million people inject drugs, and about 2.3 million (15.2%) of them are living with HIV [5]. People Who Inject Drugs (PWID) are at 35 times higher risks of acquiring and transmitting HIV and other sexually transmitted infections (STI) [1].This increased risk is not only a result of their engagement in risk behaviors such as the use of non-sterile and shared syringes and unprotected sex, but also because they are often marginalized in society, and by government policies, which reduce their access to health information and health services [2, 3, 6].

Since the 1990s, the Sub-Saharan Africa region has become increasingly vulnerable to illicit drug production, trafficking, and consumption [7]. The drug trade has played a significant role in local and national politics in countries across the continent, particularly in the East and Southern African regions. Drug use has been growing, damaging countries’ democracy and the prospects for broad-based economic development; while, public health systems are struggling to respond effectively to this growing challenge [8–10]. The overlapping crises around the world have increased the vulnerabilities of PWID and impacted the progress of global response to the HIV epidemic [11].

Mozambique, with an HIV prevalence rate of 12.5%, has one of the highest rates in the world and is among the top three countries with the highest number of new infections [12, 13]. Since 2010, the National HIV/AIDS Strategic Plan has highlighted the importance of People Who Inject Drugs (PWID) as one of the Key Populations (KP) of the HIV epidemic [13, 14]. According to the first Bio-behavioral (BBS) survey conducted in the country, half (50.1%) of PWID in Maputo, located in the south, and close to a quarter (19.9%) of PWID in Nampula, located in the north, were infected with HIV. These results indicate that PWID are the most affected key population when compared to others key population groups (31.2% in Maputo and 17.8% in Nampula for FSW; 8.2% in Maputo and 3.7% in Nampula), and in some places have four times higher HIV prevalence than the national adult population (13.2% at national level, 16.9% in Maputo province and 5.7% in Nampula province in 2015) [13]. In addition, this survey found higher levels of current hepatitis B virus (HBV) infection (as indicated by HBsAg positivity) among PWID, with a prevalence of 32.1% in Maputo and 36.4% in Nampula, and previous hepatitis C virus (HCV) infection (indicated by positive anti-HCV antibodies) at 44.6% in Maputo and 7.0% in Nampula [13–16]. Co-infection of HIV and viral hepatitis further exacerbates the vulnerability of the PWID and was estimated at 13.1% (95% CI 7.2–18.9) for HIV/HBV, 29.5% (95% CI 22.2–36.8) for HIV/anti-HCV [14].

Drug use is also associated with mental health disorders in Mozambique [16]. However, Mozambique does not have broad substance abuse services for PWID. The treatment of patients with problems associated with drug use should officially be done in the psychiatric health facilities. However, there are only two specialized psychiatric hospitals in the country, and they have very limited resources to support drug rehabilitation and treatment. Even still, PWID do not usually recognize themselves as people with mental disorders; and therefore, the location of such service is a barrier to health seeking behaviors. Stigma and discrimination toward people with mental health diseases also impede PWID from seeking help or treatment [17].

Drug injection is a major risk factor for gaps in the HIV continuum of care. PWID report long delays with initiation of antiretroviral therapy (ART), treatment retention and have a poor treatment outcomes, including viral suppression [18–20]. There are many reasons including experiences of stigma from health care providers in addition to heavy workload of service providers and long wait times. Those situations not only result in missed opportunities to treat other blood-borne and sexual transmitted infections that are also common in this population, but also results in missed opportunities to provide preventive services. Limited specific HIV prevention programs for PWID in Mozambique could be a potential reason for high HIV burden among this population. In other settings, integrated ART provision and care at specialized centers for people who use drugs (PWUD) has been documented to close these gaps in HIV care [21–23].

## Harm reduction interventions for PWID

Harm reduction is an evidence-based approach to prevent and provide treatment and care to PWID. The comprehensive package recommended by WHO and other UN agencies includes nine interventions: needle/syringe programs (NSP); Opioid Assisted Medical Treatment (OAMT); HIV testing and counseling; HIV treatment and care; condom programming for PWID and their partners; behavioral interventions; prevention and management of viral hepatitis, tuberculosis and mental health conditions; sexual and reproductive health interventions; and provision of naloxone and training on overdose prevention for the PWID community [24]. Countries that have successfully implemented and scaled up harm reduction strategies have experienced steep declines in HIV infections among PWID [25–27]. However, in low-middle-income countries, data demonstrate that those countries fail to provide PWID with access to the recommended comprehensive package of evidence-based HIV treatment and harm reduction services due to low levels of funding, competing resources, political inaction, ongoing stigmatization and marginalization among PWID, limited health services coverage, among others [22, 28, 29].

Although the greatest impact will be achieved when all interventions in the comprehensive harm reduction package are implemented together, at minimum the country should prioritize NSP, OAMT, and testing and treatment for HIV and hepatitis for PWID. After the implementation of the first Bio-behavioral survey among PWID (2013–2014), the Ministry of Health began discussions about the development of a National Harm Reduction Plan. Despite these initial steps, the Plan has yet to be put into full action. Moreover, the fifth National Strategic Plan for the HIV Response in Mozambique (2021–2025) does not recognize harm reduction as a key strategic objective, underscoring a significant gap in the country’s approach to addressing the needs of PWID.".

Traditionally in Mozambique, drug treatment has been integrated into the mental health program and psychiatric services. However, these services are under-resourced and predominantly focus on mental health care, often overlooking substance use disorders [30]. Specific social and health services, based on effective substance use education and drug treatment services, are largely limited. Even when services are accessed, PWID hesitate to disclose their drug use behavior to healthcare providers. Due to fear of stigma and discrimination and/or concerns about legal ramifications since the possession and/or use of illicit or non-medically prescribed drugs is illegal in the country (Law no 3/97, March 13, 1997).

## Tailored interventions for PWID: The drop in center

In 2018, the Médecins Sans Frontières in Mozambique (MSF), in collaboration with the Ministry of Health, FHI 360, civil society organization UNIDOS, and multisectoral authorities in Maputo City, opened the first drop-in center (DIC) for PWUD as a pilot project. This center was opened in a neighborhood with a high concentration of PWID in Maputo City. This is the first and so far, the only harm reduction intervention center in the country. The DIC in Maputo broadly serves PWID largely through peer education and outreach workers. The initiative not only provides employment opportunities to PWUD, but also provides peer-led support for prevention and treatment [20]. The initiative provides a comprehensive harm reduction package, including needle & syringe programming (NSP); OAMT with methadone; TB screening; and HIV, HCV and HBV testing and HBV vaccination. In addition, peer outreach workers work at the community level around the center providing educative information, safer injection kits, condoms and refer individuals for services and care. The results of the implementation of these strategies demonstrate that harm reduction services are most effective when offered in a friendly and non-judgmental facility environment based on their needs and easy access. The DIC creates an excellent environment for HIV testing for the PWID community because of the holistic and non-judgmental approach to serving the injecting community’s needs [20]. The project has been officially approved by the Ministry of Health for expansion.

## PWID and HIV treatment gaps

Mozambique has made significant gains in addressing its HIV epidemic. In 2013 the country implemented the HIV National Acceleration Plan which expanded treatment guidelines and vastly scaled up the availability of free ART in the country. As a result, ART provision expanded from 39 to 81% of all health facilities within the National Health System over a five-year period. However, so far, ART treatment is provided mainly through public health system. This approach brings some challenges to the public health system and health providers that have been overwhelmed by client volume, long waiting times and, and sometimes, multiple unnecessary visits to health facilities for antiretroviral drug (ARV) pick-up. Some of these have been addressed through the introduction of differentiated service delivery models, accelerated during the COVID-19 pandemic, although the impact on retention and treatment outcomes, such as viral load, has not yet been assessed.

In 2016 the MoH developed the National Integrated Guidelines for Key Populations in the Health Sector. The guidelines provide an orientation to HIV program managers and health providers on the integration of health services for KP, including PWID [13]. However, it does not include specific guidance that focuses on PWID. These challenges impact adherence to care and continuity of treatment. Less than 50% of HIV + participants from the first Bio-behavioral survey were in treatment at the time of the survey and in another study of the DIC in Maputo, less than 60% of HIV-positive PWID were linked to treatment and retained after six months [14, 20].

Additionally, despite higher health care problems, PWID also face a fragmentation of services for HIV, viral hepatitis, TB, sexual and reproductive health, and other harm reduction strategies. It is well established that the absence of quality and friendly PWID-oriented services can result in treatment avoidance or interruption [31]. This calls for the *normalization* of the provision of HIV care and treatment in specialized health care facilities outside of the routine public health system. The most effective programs aimed at reducing HIV and other health risks for PWID should co-locate HIV prevention services with other services. Not only this strategy can decrease number of visits at health facilities but also improve privacy.

## Leave no one behind: Can we “normalize” HIV treatment at specialized centers for PWID?

Effective HIV care and treatment must reflect the realities of PWID, as well as the constraints and opportunities of the social and legal contexts in which they live. Integrated care models for HIV and other diseases demonstrate a strong potential for improving treatment outcomes for PWID [3, 32–34]. Should sufficient investments be made, the expansion of DIC offers the opportunity to develop integrated models of HIV care and treatments in a specialized context. Through this model, community outreach workers can also act as frontline peer educators, who are in permanent contact with PWID to encourage them to regularly access other health services.

It is well established that early diagnosis, well-developed referral systems, and both treatment and adherence support are the key to improved health outcomes among this population [22, 27, 35]. Therefore, the decentralization of HIV prevention and treatment interventions for PWID at a DIC brings HIV services closer to them, especially in settings where there are substantial transportation costs, long waiting times or the fear of stigmatizing attitudes of health providers, all of which can have a negative impact on health seeking behaviors of this population. At the same time, efforts must still be made to raise the capacity of the current public health system to provide PWID-friendly services, free from stigma and discrimination, especially in settings where the introduction of a DIC may not be cost-effective.

## Looking ahead

Besides strong empirical evidence demonstrating the impact of specialized interventions for PWID, there are several anticipated barriers for the acceptance of these innovative health care strategies. The primary barrier will be with shifting away from the criminalization of drug use to the adoption of a public health approach. Addressing these barriers with innovative approaches will require a close collaboration with local civil society organizations, policy makers, and the acceptance of health care providers, policy authorities, and religious institutions. Health provider educational institutions as well as the Ministry of Health need to strengthen the curriculum and continuing education training of health care providers, including an enhanced focus on human rights, ethics, stigma, and discrimination. In addition, a highest priority should be given to interventions targeting children and youth, since the use of drugs in early ages increase the risk for eventual injection drug use [36, 37].

The HIV response in Mozambique, while having strong political support, is primarily dependent on external financing, with 97% of its funding coming from sources like The United States President's Emergency Plan for AIDS Relief (PEPFAR), The Global Fund, among others, and only 2% contributed by the domestic government [38], and this could be a potential reason behind the delayed introduction of specialized HIV treatment services outside of the public health care system, given that funding and political will is needed to maintain the long-term services sustainability.

Integrating ART and OAMT have the ability to improve ART access, adherence and treatment outcomes among HIV and hepatitis (B and C) co-infected PWID. Implementation of HCV treatment has been a challenge in the country. However, due to highest co-infection of HIV and hepatitis B/C it requires a renewed focus. The Ministry of Health and its partners need to address how to improve access to HCV treatment among PWID. The provision of OAMT, in combination with HCV and HIV-HCV care, could have strong implications for improved treatment uptake, adherence, and health outcomes.

Many communities of PWID are more comfortable with and are more likely to access HIV services outside the formal public health sector where services are provided in safe and convenient places and PWID are less likely to experience stigma and discrimination [20, 39]. The DIC provides “one-stop shop” strategy for addressing complex healthcare needs including a variety of treatment interventions such as needle distribution, OAMT, HIV counseling and prevention, among others. The effectiveness of combining HIV treatment with OAMT is well documented [40, 41]. This environment is not only convenient for PWID, but also results in multiple needs being addressed more effectively.

In 2021, the MOH released a draft of a mobile clinic strategy that provides comprehensive clinical and psychosocial support for KP, and authored partners to start implementing these decentralized services to KP hotspots. This intervention has been documented to support retention and adherence among PWID. This strategy can also support the connection between the community and the DIC.

## Conclusion

Mozambique has one of the highest HIV prevalence estimates in the world. Refocusing and targeting responses to the highest-risk groups is essential for shaping more effective HIV interventions and achieving epidemic control. A people-centered approach, recognizing the unique realities of PWID, is likely to foster trust and engagement in HIV preventive and treatment services and ultimately improve health outcomes among this population.

It is difficult to predict future trends in drug consumption, although the potential increase in drug consumption cannot be ignored. It will thus be crucial in the next years for the government to work toward implementing interventions to decrease HIV and viral hepatitis new infections, which should include equitable and equal access to HIV services and breaking down barriers required to achieve the new Global Targets set by UNAIDS. Integrating HIV care and treatment in specialized centers for PWID that provide primary health care, STI prevention and care, substance abuse and psychiatric services may ensure the equitable advancement toward ending AIDS as a public health threat for all.

## Data Availability

Not applicable.

## Abbreviations

UNAIDS: The Joint United Nations Program on HIV/AIDS
STI: Sexually transmitted infection
AIDS: Acquired immunodeficiency syndrome
HIV: Human immunodeficiency virus
PWID: People who inject drugs
DIC: Drop-in-center
BBS: Bio-behavioral survey
HBV: Hepatitis B virus
HCV: Hepatitis C virus
ART: Antiretroviral therapy
PWUD: People who use drugs
NSP: Needle/syringe programs
OAMT: Opioid assisted medical treatment
PEPFAR: The United States President's Emergency Plan for AIDS Relief

## Declarations

### Disclaimer

The views and opinions in this publication are those of the authors and do not represent the view of authors affiliation institutions.
